# Combination Filling

**Published:** 1899-09

**Authors:** F. B. Spooner

**Affiliations:** Brooklyn.


ARTICLE VII.

### COMBINATION FILLING.

F. B. SPOONER, D. D. S. BROOKLYN.

For the last two years I have been experimenting to find the "ideal" filling so long talked about in our meetings. I do not mean to say it is found, but will give the result of my efforts.

We all know the essentials of the "ideal." Gold costs too much in time and material. Amalgam shrinks, discolors, and is a conductor. Cement is perishable, but ideal in all other respects. Could we get cement to be as lasting as amalgam, what a stride it would be.

The method which I shall describe occurred to me while I studied the manner by which the crumbling of the river bank at Wheeling is prevented. There the approach of the river is paved with stone mixed with the soft earth. This is done so that the wash of the river at high mark, and the passage of vehicles shall not wear through. The same thing is seen in a chimney exposed to long years of weather. If it were made of mortar, it would soon succumb, but as it shows, the mortar is eaten out between the bricks only to a degree.

The conclusion was reached that if I could mix my cement with metal cobblestones, the wash of saliva and other causes of disintegration could be lessened.

Mixing my cement with various quantities of alloy, I finally hit on this. On a pad was placed two equal piles of material, one amalgam, the other cement; also acid Q. S, The alloy was *first* mixed with the liquid. Why? To be sure that the metal becomes thoroughly coated with the liquid. No fear that the cement would finally mingle with the acid, and also form perfect union with the metal. Next mix the zinc powder with the wet alloy, working it up to a tolerably stiff mass. It may be said, by the way, that there is *much* in spatulating cement. It should be so done that all parts be thoroughly incorporated.

A lesson may be taken from a laborer who mixes mortar with hair, sand and lime. There should be a chopping, grinding, pressing, rotary motion, all at one and the same time.

The mass hardens quickly, and so has to be handled with expedition. Sometimes I put a part of the softer mix into the cavity, following with the stiffer. In this way I get more adhesion with the one, and strength with the last. A surplus is put in while an instrument dipped into the powder can be used to contour the filling to the edges. A matrix is also used slightly oiled, when it is desirable to do so.

Stress is laid on first mixing the alloy with the liquid. At the risk of being tedious, will say that it is to ensure the cement going into the intermolecular spaces, formed by the alloy as the molecules. As the alloy is first coated with the fluid, it is certain that the cement will adhere to the metal, leaving no soft spots. Hence the great point is "if you use it, use it right." Moreover, I speak of the way, as already a method has been advocated of mingling cement and alloy which has been briefly told of in an Atlanta magazine. I read of the same while pursuing my researches.

I do not think that alloy is necessary for the work; on the contrary, tin fillings would be preferable on the ground of cheapness, and that discoloration would not occur,

We have for this filling the following advantages:

A non-conductor, better than amalgam.

Adhesive, holding together frail walls,

Result, a filling that will not expand, contract, or discolor; a filling that will not wash or wear away as soon as cement.

As this combination has not been in use more than two years, data as to its permanence is not obtainable, although up to date the results are favorable. Time must show how far it falls short of amalgam in resistance to mastication. Still this feature is far from *all* in fixing the value of a filling. Those fatal margins will always menace an amalgam. As a rope "is only as strong as its weakest point," so a filling with this weakness may fail in the long run against a material with tight edges, but less ability to resist attrition.

Thls much seems fair to conclude that (while I am far from desiring to discard amalgam) there are times when it is not indicated, and that if only the life of a cement filling is prolonged, it is a step onward.—*Items of Interest.*

